# Letter from the Editor in Chief

**DOI:** 10.19102/icrm.2018.090308

**Published:** 2018-03-15

**Authors:** Moussa Mansour


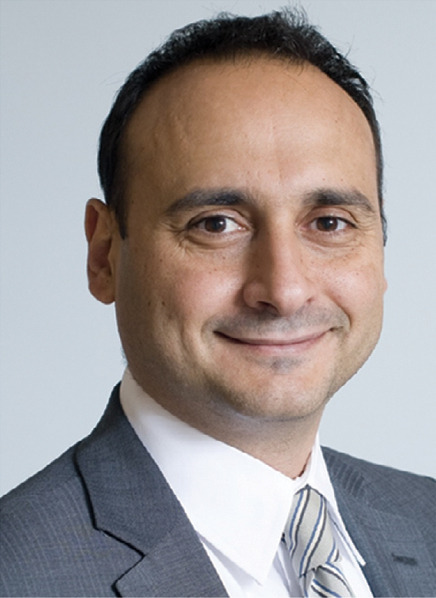


Dear Readers,

Catheter ablation for atrial fibrillation (AF) has been performed since the late 1990s. A large number of studies have been conducted to investigate and compare different aspects of ablation, including various energy sources and ablation strategies and the use of new technologies. Until recently, most of the studies in the field of AF have demonstrated soft endpoints including time to first recurrence or improvement in quality of life, rather than solid outcome measures such as effect on mortality.

However, in the past year, the results of two landmark multicenter randomized clinical trials have been published, with both demonstrating improved survival with AF ablation versus medical treatment in patients with congestive heart failure. The first was the Ablation vs. Amiodarone for Treatment of Atrial Fibrillation in Patients With Congestive Heart Failure and Implanted ICD/CRTD (AATAC) study,^1^ which showed that catheter ablation of AF is superior to amiodarone use in achieving freedom from AF at long-term follow-up and in reducing unplanned hospitalizations and mortality in patients with heart failure and persistent AF. In this study, the hospitalization rate was 31% in the ablation group and 57% in the amiodarone group. More importantly, there was a survival benefit associated with ablation, with observed mortality rates of 8% with ablation and 18% with medical treatment. These findings were corroborated in the Catheter Ablation for Atrial Fibrillation with Heart Failure (CASTLE AF) study,^2^ which determined that catheter ablation of AF is associated with improved survival and reduced hospitalization rates for worsening heart failure when compared with medical treatment.

The designs and findings of these two studies represent a major step forward in the field of AF ablation. The results of the notable CABANA study (Catheter Ablation vs Anti-arrhythmic Drug Therapy for Atrial Fibrillation Trial), will be presented later this year. I believe that more studies will also be conducted in the future to investigate the effects of AF ablation on survival in other patient populations, which will lead to significant growth in the number of AF ablations.

I hope you enjoy this issue of *The Journal of Innovations in Cardiac Rhythm Management.*

Sincerely,


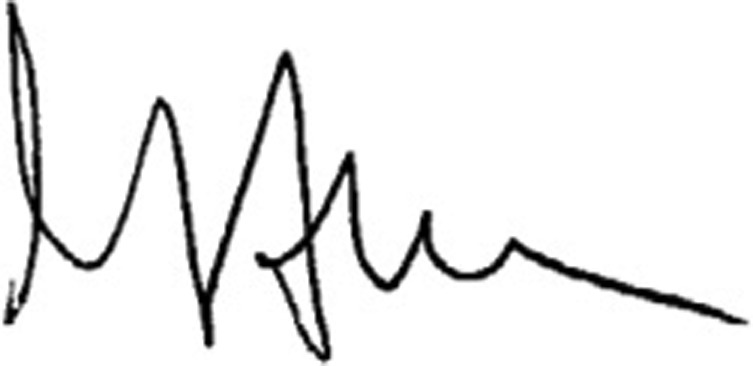


Moussa Mansour, md, fhrs, facc

Editor-in-Chief

The Journal of Innovations in Cardiac Rhythm Management

MMansour@InnovationsInCRM.com

Director, Atrial Fibrillation Program

Jeremy Ruskin and Dan Starks Endowed Chair in Cardiology

Massachusetts General Hospital

Boston, MA 02114
